# Automatic transition from AAIR to VVI mode: the impact of capture loss in His bundle pacing

**DOI:** 10.1093/ehjcr/ytaf018

**Published:** 2025-01-21

**Authors:** Sujoy Khasnavis, Samer Saouma, Jay Gross

**Affiliations:** Department of Cardiology, Montefiore Medical Center, 3415 Bainbridge Avenue, Bronx, NY 10467, USA; Department of Cardiology, Northwell Health, 210 E 64th St, New York, NY 10065, USA; Department of Cardiology, Montefiore Medical Center, 3415 Bainbridge Avenue, Bronx, NY 10467, USA

## Clinical vignette

**Figure 1:**
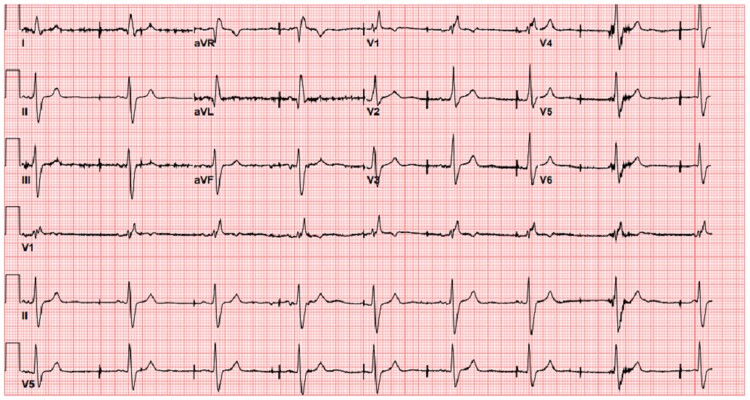
Admission ECG.

A 74-year-old male with sinoatrial node dysfunction was admitted for a syncopal episode. He had a dual-chamber Medtronic Adapta ADDR01 pacemaker implanted 10 years earlier. The device was programmed to AAIR<=>DDDR pacing mode with the right ventricular lead in His position. Pacing and sensing parameters were normal after placement. Admission electrocardiogram (ECG) is shown in *Figure 1* below recorded at 25 mm/s. The patient was not on any antiarrhythmic drugs prior to the observed ECG changes. Intracardiac reading and marks were not available during pacemaker interrogation at the time of the ECG recording.

### Questions 1

**What rhythm is shown in the ECG?**

- Normal sinus rhythm
- Sinus bradycardia with variable pacemaker interval
- Accelerated idioventricular rhythm
- Sinus bradycardia with fixed pacemaker interval
- Normal sinus rhythm with bifasiscular block

**Answer: B**

Choices A and E are incorrect since the rhythm shown is in bradycardia. Choice C is incorrect since idioventricular rhythm is asymptomatic and seen after infarction, characteristics not seen here. Choice D is incorrect since the pacing interval changes with the QRS complexes. Essentially, the device was in VVI backup mode at a basal rate of 65 beats per minute before malfunction. Prior to VVI, there was no ventricular pacing since the device was in effective AAIR to DDDR programming. VVI sensitivity was 2.0 mV, and V refractory period was 230 ms. Atrioventricular (AV) delays were not applicable in either mode.

### Questions 2

**What is the abnormality in pacemaker function?**

- Atrial non-capture with appropriate atrial and ventricular sensing
- Ventricular under-sensing
- Normal pacemaker function
- Ventricular non-capture with appropriate ventricular sensing
- Atrial non-capture with appropriate atrial sensing

**Answer: D**

Choices A and E are incorrect as there is no atrial pacing or sensing. Choice B is incorrect as pacer spikes vary with QRS. Choice C is incorrect due to abnormal pacemaker function. Although the device was programmed to DDDR mode, the end of life VVI mode set at 65 b.p.m. was ineffective without His capture. Replacing battery alone would ensure pacing through the atrial lead but a ventricular lead was implanted to protect from AV conduction abnormalities. Atrial threshold measurement was not possible in back up mode. Ventricular capture was not possible even at maximal output due to lack of capture.

### Questions 3

**What is the most likely cause of pacemaker failure and resulting symptoms?**

- Compromise in the physical integrity of leads
- Normal pacemaker function
- Compromise in His bundle pacing
- Pulse generator failure
- Compromise in atrial pacing

**Answer: C**

Choices A, B, and D are incorrect as pacemaker activity is abnormal. Choice E is incorrect as loss of atrial pacing alone would not cause symptoms in DDDR mode. Due to rising thresholds with ineffective His pacing, pacer spikes are seen after the preceding QRS complexes consistent with ventricular non-capture. Ventricular sensing, however, is intact due to variability in the pace–pace interval and QRS complexes not falling in the sensing refractory period. Thresholds can rise over time in the absence of physical compromise in lead integrity, which was observed in a case where AAIR switched to VVI near recommended replacement time.^1^

## Supplementary Material

ytaf018_Supplementary_Data

## Data Availability

The data underlying this article are available in the article and in its online Supplementary material.

## Notes

**Consent:** Written and verbal consent for this publication was obtained from the subject.

**Funding:** No funding obtained for this report.
