# Supplementary material for: Automatic transition from AAIR to VVI mode: the impact of capture loss in His bundle pacing
Source: Eur Heart J Case Rep. 2025 Jan 21;9(2):ytaf018. doi: 10.1093/ehjcr/ytaf018 (PMC11799935; doi:10.1093/ehjcr/ytaf018)
Supplement: ytaf018_Supplementary_Data [file ytaf018_supplementary_data.zip › Supplemental 1.docx]

**Supplementary Figure 1**

**Figure 1.** Anteroposterior chest x ray. Note the dual chamber pacemaker with RV lead positioned at the typical His location
