# Supplementary material for: Automatic transition from AAIR to VVI mode: the impact of capture loss in His bundle pacing
Source: Eur Heart J Case Rep. 2025 Jan 21;9(2):ytaf018. doi: 10.1093/ehjcr/ytaf018 (PMC11799935; doi:10.1093/ehjcr/ytaf018)
Supplement: ytaf018_Supplementary_Data [file ytaf018_supplementary_data.zip › Supplemental 2.docx]

**Supplementary Figure 2**

**Figure 2A: Pre-op ECG.** Admission ECG with original pacemaker at battery end of life. Pacer

spikes appear long after the prior QRS. Notable variability in the interval between consecutive

pacer spikes.

**Figure 2B: Post-op ECG.** ECG after dual chamber pulse generator and pacing lead placements in RVOT. Notable return to AAIR and resolution of bradycardia.
